# Supplementary material for: Integrating Biochemical and Computational Approaches Reveal Structural Insights in Trastuzumab scFv-Fc Antibody Engineering
Source: Biomolecules. 2025 Apr 22;15(5):606. doi: 10.3390/biom15050606 (PMC12108635; doi:10.3390/biom15050606)

Supplemental Material

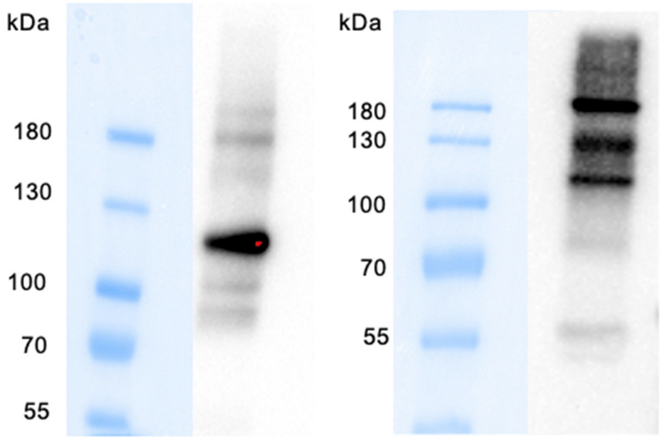

Supplemental Figure S1. Non-reducing SDS-PAGE of the WT scFv-Fc constructed in the V_H-V_L (left panel) and V_L-V_H (right panel) orientations. Western blot original images can be found in Supplementary Materials.

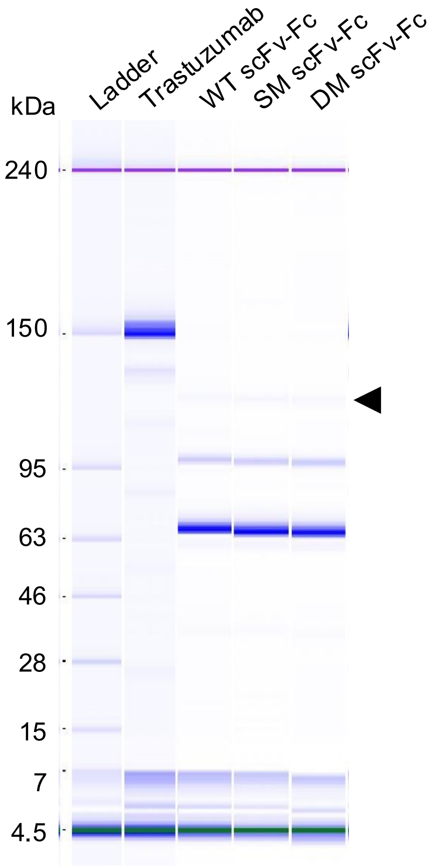

Supplemental Figure S2. Agilent Bioanalyzer 2100 analysis of degraded engineered scFv-Fc antibodies. Initial attempt to purify scFv-Fc using Protein A Fc-affinity column. Black arrow indicates place of expected band for intact scFv-Fc dimers.

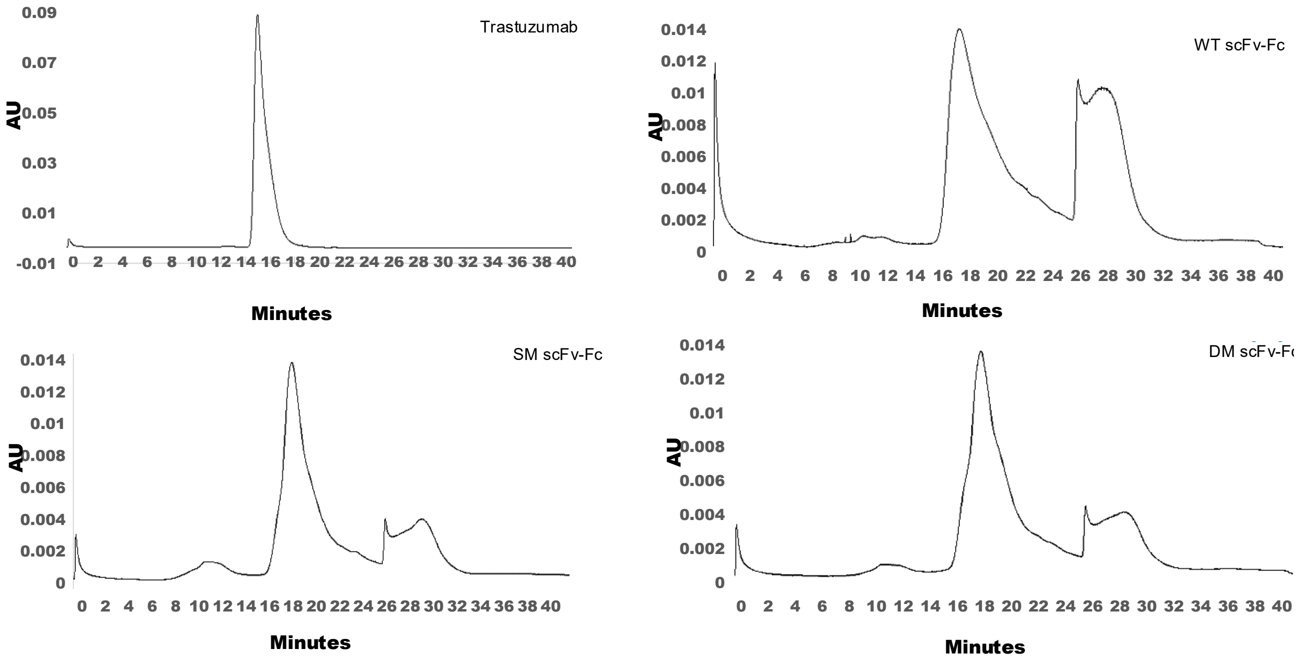

Supplemental Figure S3. SEC-HPLC elution profiles of scFv-Fc antibodies. The elution times of purified trastuzumab and the scFv-Fc counterparts monitored at 280 nM.

	1	10	20	30	40	50	60
Heavy chain<br>Light chain	EVQLVESGG<br>DIQMTQSPS	GLVQPGGSLR<br>SLSASVGDRV	LSCAASGFNI<br>TITCRASQDV	KDTYIHWVRQ<br>NTAVAWYQQK	APGKGLEWVA<br>PGKAPKLLIY	RIYPTNGYTR<br>SASFLYSGVP	YADSVKGRFT<br>SRFSGSRSGT
	70	80	90	100	110	120	
Heavy chain<br>Light chain	ISADTSKNTA<br>DFTLTISSLQ	YLQMNSLRAE<br>PEDFATYYCQ	DTAVYYCSRW<br>QHYTTPPTFG	GGDGFYAMDY<br>QGTKLEIK	WGQGTLVTVS	S	

	215	220	230	240	250	260	270
Hinge-Fc	VEPKS	CDKTYTCPPC	PAPELLGGPS	VFLFPPKPKD	TLMISRTPEV	TCVVVDVSHE	DPEVKFNWYV
	280	290	300	310	320	330	340
	DGVEVHNAKT	KPREEQYNST	YRVVSVLTVL	**H**QDWLNGKEY	KCKVSNKALP	APIEKTISKA	KGQPREPQVY
	350	360	370	380	390	400	410
	TLPPSREEMT	KNQVSLTCLV	KGFYPSDIAV	EWESNGQPEN	NYKTTPPVLD	SDGSFFLYSK	LTVDKSRWQQ
	420	430	440	450			
	GNVFSCSVMH	EALHN**H**YTQK	SLSLSPGKHH	HHHH			

H310 and H435 in bold.

Supplemental Figure S4. The amino acid sequence of the heavy and light chains for scFv-Fc antibodies.

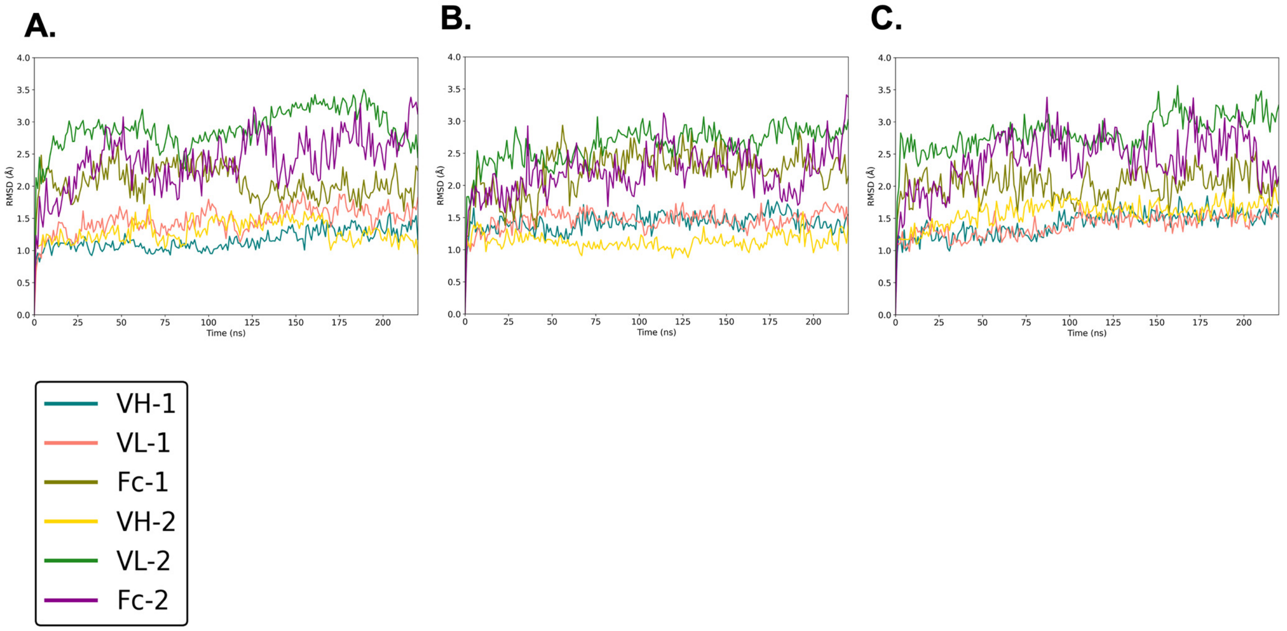

Supplemental Figure S5. The domain-resolved RMSD for the A) WT, B) SM, and C) DM scFv-Fc antibodies. Each trace depicts the RMSD of a specific domain (color coded and numbered for either chains 1 or 2) calculated over the course of the 220 ns simulation.

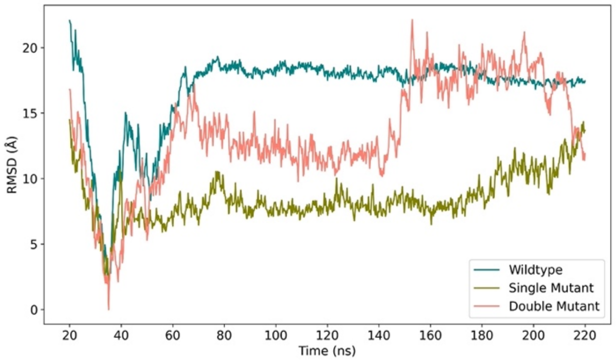

Supplemental Figure S6. RMSD plots from MD simulations for the scFv-Fc antibodies calculated after removing the first 20 ns.

Original images

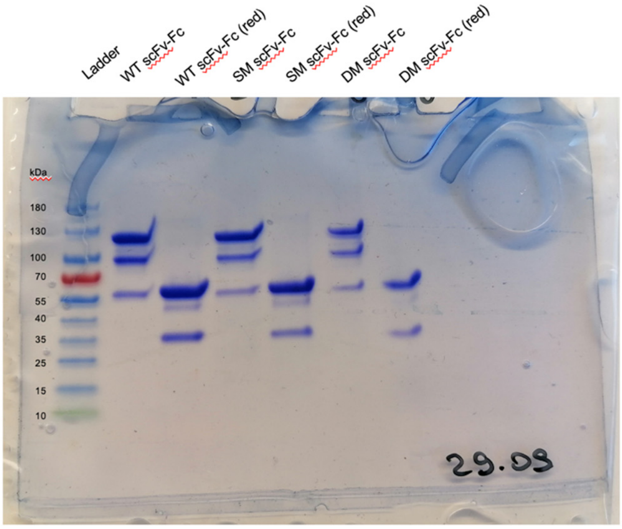

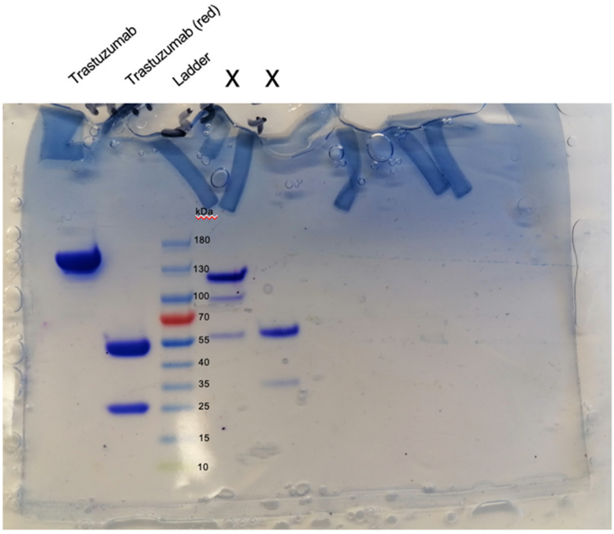

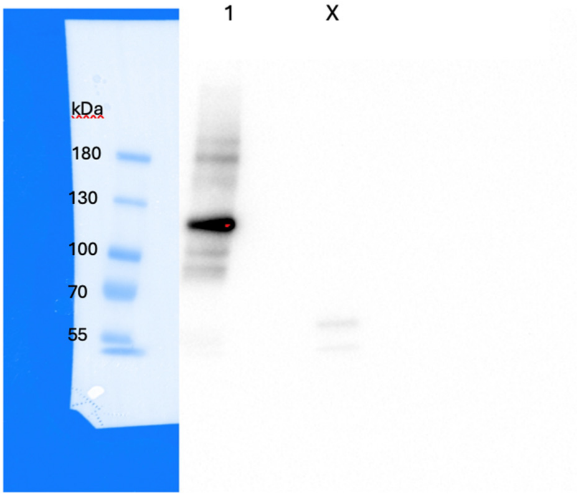

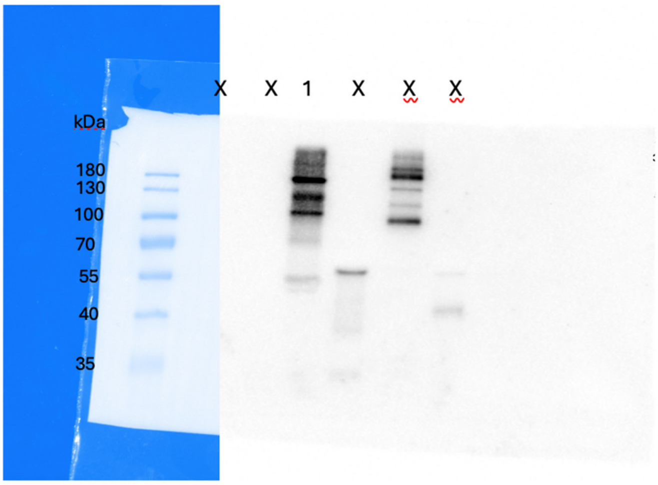

Supplement: Supplementary file 1 [file biomolecules-15-00606-s001.zip › biomolecules_supplemental material_R1.pdf]
